# Intraosseous schwannoma of the glenoid: case report and literature review

**DOI:** 10.1051/sicotj/2020048

**Published:** 2021-01-08

**Authors:** Pieter Reyniers, Hazem Wafa, Friedl Sinnaeve, Philippe Debeer, Raf Sciot

**Affiliations:** 1 University Hospitals Leuven Campus Gasthuisberg Herestraat 49 3000 Leuven Belgium

**Keywords:** Schwannoma, Neurilemmoma, Intraosseous, Glenoid

## Abstract

Intraosseous schwannomas represent an extremely rare subgroup of schwannomas, accounting for <1% of all primary bone tumors. They mostly occur in the mandible, the maxilla, the sacrum, and they are also seen in long bones. We herein report a rare presentation of an intraosseous schwannoma in the glenoid of a 49-year-old patient. She complained of shoulder pain and was referred to the orthopaedic oncologist after detection of a suspicious lesion on imaging. Biopsy revealed benign spindle cells and immunohistochemistry was positive for S100. Because of the rarity of these intraosseous schwannomas it is important to recognize their radiological and histological features and make a differential diagnosis with other lytic tumors. Only if these characteristics are recognized, correct treatment can be given with definite curettage and bone grafting and correct follow-up with avoidance of unnecessary adjuvant therapy.

## Introduction

Schwannomas are benign nerve sheath tumors, comprising approximately 5% of all benign soft tissue tumors. They arise from Schwann cells that form the myelin sheath, insulating the nerve [1]. There is a predilection to affect sensory nerves and as such may be painful. There is no sex predilection and they can occur at any age [1, 2]. About 90% of these detections are sporadic cases and ten percent is in association with syndromes like neurofibromatosis (in which vestibular schwannomas are a hallmark) or schwannomatosis (sporadic or familial) or in the very rare setting of the Carney complex [3, 4]. The chance of malignant transformation to malignant peripheral nerve sheath tumor (MPNST) is small but has been reported. Different histological variants of schwannoma have been described such as classic, epithelioid, cellular, microcystic, and neuroblastoma-like types, but with limited clinical significance [3]. In this case report, we like to focus on intraosseous schwannoma which is an extremely rare variant. This intraosseous presentation of schwannomas accounts for <1% of all primary bone tumors and Ida et al. estimated a frequency of approximately 0.1% [4, 5]. We present a rare case of an intraosseous schwannoma in the glenoid.

## Case presentation

A 49-year-old female patient presented to our orthopedic unit with left shoulder pain for 3 months. She had a history of cervical scoliosis. The pain had been increasing progressively with exertion and was situated posteriorly on the scapula and trapezius muscle, but also in the deltoid region spreading to the distal part of the arm. She was complaining of paresthesia in the hand, without any evidence for a specific dermatome delineation. She experienced the exact same complaints 2 years ago. Magnetic resonance imaging (MRI) of the cervical spine was performed at that time together with electromyography in another hospital, which did not show any explanation for her pain. Eventually, her complaints improved with acupuncture. On physical examination in the outpatient clinic, there was minimal tenderness over the deltoid muscle. The skin showed no swelling, erythema, or warmth. She had a full painless range of motion of the shoulder and elbow joints. There was no evidence of lymphadenopathy. Neurological examination of the cervical spine and upper limb was normal. Laboratory results did not reveal any obvious abnormality.

**Figure 1:**
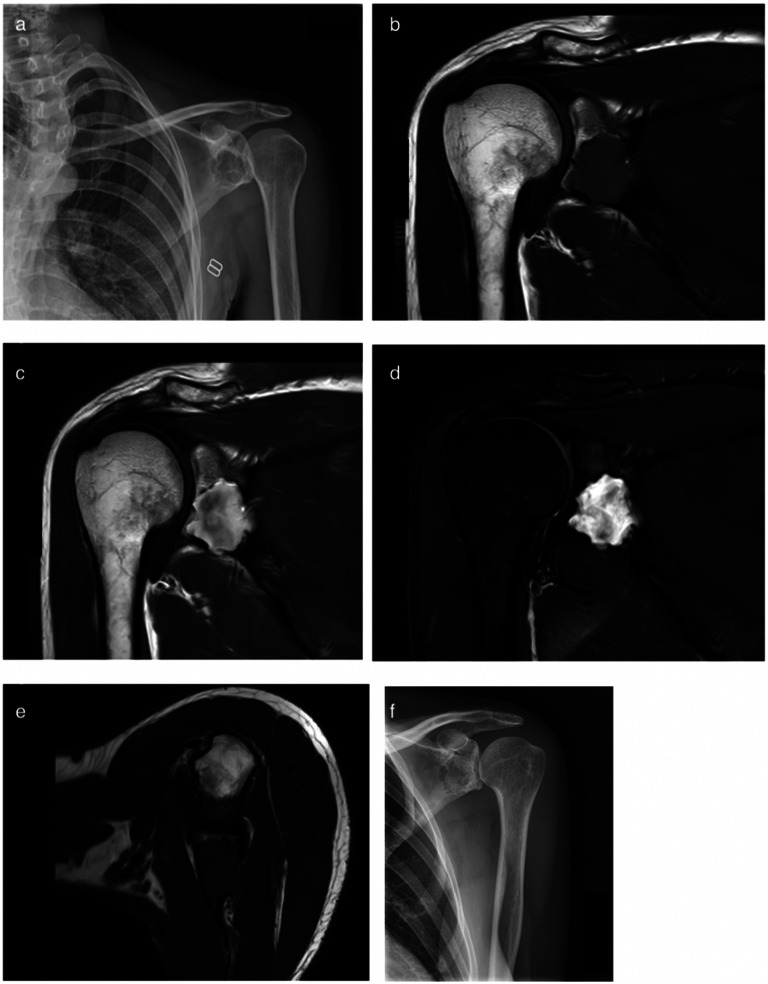
(a) X-ray shows osteolytic lesion in the glenoid with sclerotic border and trabeculation. (b and c) Frontal images in T1WI show an intraosseous lesion with clear cortical breach inferiorly and discrete soft tissue expansion outside the glenoid. There is contrast enhancement after administration of gadolinium. (d) There is hyperintense signal in T2WI. (e) Axial images also show an isointense signal to muscle tissue and cortical breach laterally. (f) No recurrence is seen on X-ray after 12 months.

Conventional plain radiographs demonstrated a well-defined osteolytic lesion in the left glenoid with a thin sclerotic rim. Some trabeculation could be noticed. Subsequent MRI with gadolinium contrast of the left shoulder revealed a lobulated and expansile bone tumor in the inferior 2/3 of the glenoid measuring 26 × 24 × 17 mm. The posterior glenoid cortex had important thinning with a focal breach. The tumor was isointense to skeletal muscle on T1-weighted images (T1WI) and more heterogeneous hyperintense with some intermediate and hypointense signals on T2-weighted images (T2WI). There was heterogeneous contrast enhancement corresponding to the hyperintense zones in T2WI. There was no perilesional edema (Figure 1).

**Figure 2:**
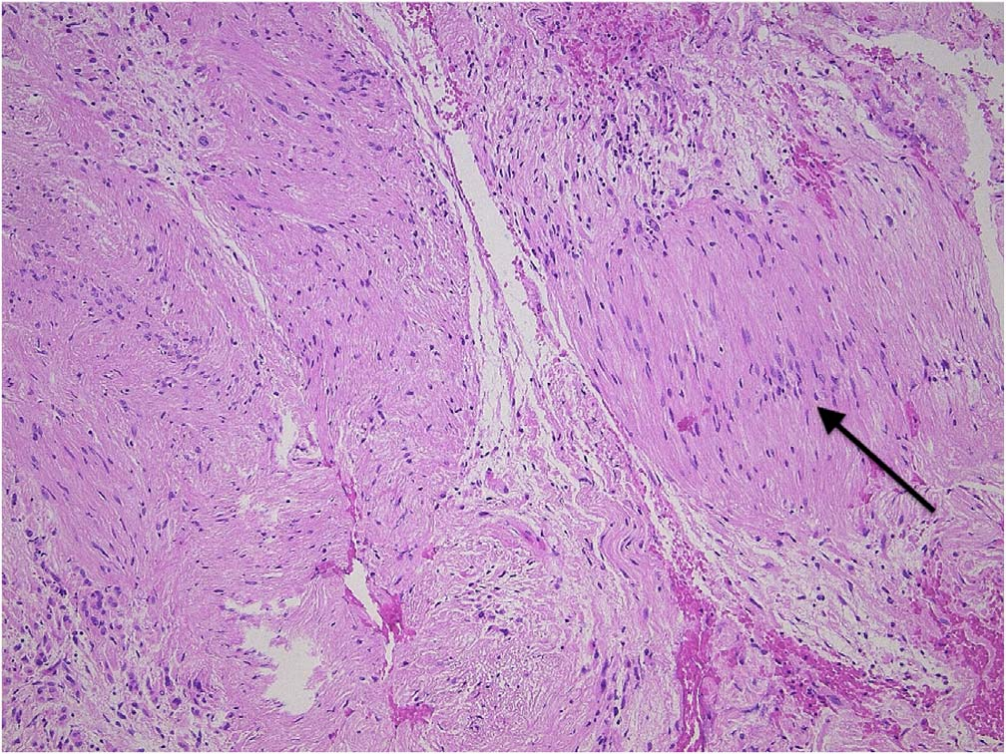
Typical picture of a schwannoma, showing the nuclear palisading (arrow).

The patient was discussed at the multidisciplinary bone and soft tissue tumor meeting and it was thought that the appearances of this lesion on the plain X-rays and MRI scan are very non-specific. It was therefore advised to obtain ample tissue through an open biopsy for the histopathological assessment of the lesion. A posterior approach was used in the lateral decubitus, with a 5 cm transverse incision along the lateral part of the scapular spine. The deltoid was detached from the exposed part of the scapular spine and distally retracted. An intramuscular approach through the infraspinatus muscle into the glenoid lesion was used. The suprascapular nerve was identified and retracted. There was no intra-articular involvement and as such the capsule was left intact. Samples were taken using a Jamshidi^®^ needle (BD, Franklin Lakes, USA) and sent for histopathological analysis and cytogenetic examination. This showed benign features with spindle cells and immunohistochemical staining for S100 protein was diffusely positive (Figure 2). The diagnosis of intraosseous schwannoma was confirmed. She was scheduled 2 weeks after the biopsy for an extended curettage and bone grafting with lyophilized allograft, using the same approach. The deltoid muscle was reattached to the scapular spine using transosseous sutures with FiberWire^®^ (Arthrex, Munich, Germany). Physiotherapy was started the day after the operation and a sling was given for comfort.

The patient gradually regained mobility and pain subsided. No recurrence was seen 12 months after surgery (Figure 1).

## Discussion

### Incidence and previous reports

The first histologic description of a neurilemmoma, later called a schwannoma, was made by Verocay in 1908 and elaborated upon by Stout in 1935 [6]. In the 1970’ only 48 intraosseous cases of schwannomas had been described [7]. Until today, less than 200 cases have been reported [2, 4, 7–12]. There is no sex predilection and the second and third decade is the most frequent time of diagnosis [5]. More than half of these cases (56% as reported by Suzuki et al. in a literature review) are diagnosed in the maxillofacial skeleton [2, 9]. This is because of the abundance of sensory nerves in this region. Soft tissue schwannomas concordantly have an incidence of up to 48% in the head and neck region [9]. There are 2 previous reports of an intraosseous schwannoma in the scapula. One report in 2014 was an intraosseous tumor of the scapula extending to the glenoid in a 42-year-old female patient complaining of shoulder pain for 4 years. There was a larger extraosseous component than the patient in our study [12]. Another intraosseous schwannoma of the glenoid was reported in 1967 in a 24-year-old female patient with shoulder pain for 3 years and muscle atrophy on clinical examination [13].

### Origin

The discussion arises about the origin of these intraosseous schwannomas. First of all, one of the possibilities is that they are located along a sensory nerve in an intraosseous canal. This is an explanation of why these tumors frequently occur in the mandible, the maxilla, and the sacrum. Bones like these have many nerves traversing in canals or foramina. In a long bone, schwannomas can occur in a nutrient canal [14]. This results in a dumbbell configuration with the destruction of the affected bone [10, 15]. Giant sacral schwannomas have been described [16]. The suprascapular nerve runs in the suprascapular notch. In this case, the inferior part of the glenoid was involved with no evidence of origin in the scapular notch. No dumbbell configuration was observed.

Secondly, an intraosseous schwannoma can originate in the medullary cavity from an intraosseous sensory nerve. Schwannomas show a predilection for myelinated, sensory nerves and most intraosseous nerves are non-myelinated and participate in vasomotor functions. Intraosseous sensory nerves are scarce which can be the explanation why intraosseous schwannomas are very rare. Intraosseous sensory nerves access the medullary cavity in a nutrient canal, creating an intramedullary tumor with characteristics of a true primary bone tumor. This is a reasonable hypothesis for intraosseous tumors involving the long bones. To support this theory, an association was found between the position of an intraosseous schwannoma and the position of nutrient vessels in the distal humerus, proximal ulna, distal radius, and the fibula as examined by Suzuki et al. [2]. Concerning the scapula, nutrient foramina were recently described with an average number of 5.3 nutrient foramina per scapula. Most nutrient foramina however were found in the supraspinous fossa (29.7%) and were least present in the peri-glenoid area (17.7%). The presence of a nutrient canal in the glenoid region in this patient cannot be ruled out [17].

Finally, an outside-in tumor growth pattern has also been suggested in which schwannomas destruct the bone from the outside. They originate from periosteal sensory nerves and give periosteal reaction [10, 11]. It is possible that in this patient the tumor originated from the inferior posterior glenoid periosteum and protruded into the bone, but no periosteal reaction was seen.

### Pathology

Histologic features, in this case, were consistent with findings in the literature. Intraosseous schwannomas are microscopically comparable to soft tissue schwannomas with alternating cellularity of Antoni type A and B areas. The cellular area of this lesion may be confused with sarcoma, but no other malignant features such as atypia, mitotic figures, or necrosis are seen in schwannoma [18]. Immunohistochemical staining performed for S100 protein was strongly positive within the nuclei and cytoplasm of the neoplastic spindle cells.

### Diagnosis

Schwannomas are slowly growing lesions. Some patients have some vague pain for several years. There can be some local muscle tenderness. X-rays show benign osteolytic lesion with sclerotic borders, trabeculation, cortical thinning or breach with no central calcification [10, 11]. These specific findings also match other benign bone tumors like cysts, giant cell tumors, chondroblastoma, fibrous dysplasia, chondromyxoid fibroma, neurofibroma, or non-ossifying fibroma. Malignant peripheral nerve sheath tumor (MPNST) and malignant fibrous histiocytoma usually grow more rapidly, but one must be careful to exclude these tumors before pathology results are known [4, 10, 12]. Subsequent MRI can further confirm a benign tumor and can visualize extraosseous involvement when present. T1WI is isointense to muscle and T2WI can be homogeneously or like in this case heterogeneously hyperintense to fat. When the histologic image is very typical, additional immunohistochemical staining for S100 can further confirm the diagnosis. In this study, another antibody staining like Ki-67 is not used because of the lack of additional diagnostic value. When surgery is performed, primary schwannomas are rarely encapsulated, unlike soft tissue schwannomas [4].

### Treatment and prognosis

Intraosseous schwannoma can successfully be treated by intralesional curettage and filling of the tumor cavity with either bone graft, PMMA, or bone graft substitute, or *en bloc* open excision for larger lesions [19]. We have opted for filling the bone cavity after curettage with bone graft rather than PMMA as this is more biological with less chance of developing arthritic changes in the joint in the long term. No malignant transformation has ever occurred in intraosseous schwannomas [2, 4, 14]. Two cases of recurrence have been described, which probably can be attributed to incomplete resection [4, 8].

## Conclusion

Intraosseous schwannomas are rare tumors with benign features. They grow slowly and cause local tenderness. Careful radiographic interpretation is necessary and diagnosis is made after open biopsy and examination by the pathologist. They grow within canals and foramina, appear in the medullary cavity originating from an intraosseous sensory nerve, or grow from outside in. The common treatment is curettage and bone grafting. The patient can be reassured that no malignant transformation has ever been seen in intraosseous schwannomas. Recurrence is very rare and can be due to incomplete resection.

## Conflict of interest

No benefits in any form have been received or will be received from a commercial party related directly or indirectly to the subject of this article.
